# STAT5 and CD4 ^+^ T Cell Immunity

**DOI:** 10.12688/f1000research.9838.1

**Published:** 2017-01-11

**Authors:** David L. Owen, Michael A. Farrar

**Affiliations:** 1 Center for Immunology, Masonic Cancer Center, and Department of Laboratory Medicine and Pathology, University of Minnesota, Minneapolis, MN, 55455, USA

**Keywords:** STAT5, T-cell development, T-cell function

## Abstract

STAT5 plays a critical role in the development and function of many cell types. Here, we review the role of STAT5 in the development of T lymphocytes in the thymus and its subsequent role in the differentiation of distinct CD4 ^+^ helper and regulatory T-cell subsets.

## Introduction

The transcription factor STAT5 is expressed in all lymphocytes and plays a key role in multiple aspects of lymphocyte development and function. STAT5 is a modular transcription factor that consists of an N-terminal domain that allows for homotypic interactions and tetramerization ^1^, a DNA binding domain, an SH2 domain involved in recruitment to phosphorylated receptors and ultimately homodimerization, and a C-terminal transactivation domain ^2^. STAT5 was initially identified as a transcription factor activated by prolactin in mammary gland epithelial cells ^3, 4^. Subsequent studies identified STAT5 binding activity in T cells ^5^, and it was later established that STAT5 was expressed in multiple cell types and activated by a number of cytokines, including the common gamma chain (γc)-dependent cytokines interleukin 2 (IL2), IL4, IL7, IL13, and IL15 ^6^ as well as a number of γc-independent cytokines, including thymic stromal lymphopoietin (TSLP), granulocyte-macrophage colony-stimulating factor (GM-CSF), and IL27 ^7– 11^. Molecular characterization of the *Stat5* gene demonstrated that *Stat5* was encoded by two closely linked genes that encoded STAT5a and STAT5b ^12– 14^. These two genes are likely the result of gene duplication and are highly homologous. Initial studies showed that STAT5a and STAT5b bound to a similar DNA core motif, although there were subtle differences in their DNA binding preferences ^15^. Subsequent chromatin immunoprecipitation followed by massively parallel DNA sequencing (chromatin immunoprecipitation sequencing [ChIP-Seq]) studies suggest that there may be differences in the subsets of genes bound by STAT5A and STAT5B ^16, 17^. However, these two transcription factors appear to be functionally redundant if expressed at similar levels ^18^. Substantial work has focused on the role of STAT5 in both lymphocyte development and function. These studies have clearly established a critical role for STAT5 in early T-cell development and pointed to critical functions for STAT5 in distinct T-cell subsets. Here, we will briefly review the role of STAT5 in T-cell development and then focus on advances in our understanding of the role that STAT5 plays in the differentiation of distinct T-cell subsets.

## STAT5 in T-cell development

The observation that STAT5 is activated by multiple cytokines in T cells suggested that it might play a critical role in the development or function (or both) of these cells. Disruption of *Stat5a* or *Stat5b* genes alone resulted in relatively modest phenotypes; for example, *Stat5a ^-/-^* mice had defects in mammary gland development and lactation while *Stat5b ^-/-^* mice had defects in response to growth hormone in male mice and natural killer cell proliferation ^19, 20^. To determine whether combined deletion of *Stat5a* and *Stat5b* might result in more profound immunodeficiencies, subsequent studies deleted the first coding exons of both *Stat5a* and *Stat5b*. This intervention resulted in the production of truncated forms of STAT5a and STAT5b that acted as functional hypomorphs. These mice too had surprisingly mild defects in lymphocyte development, although T cells were grossly dysfunctional, as they could no longer proliferate in response to IL2 ^21, 22^. Subsequent studies using mice expressing a constitutively active form of STAT5b suggested that STAT5 might play a more critical role in lymphocytes than suggested by the studies of STAT5 hypomorphs. These mice exhibited significant expansion of progenitor B cells, CD8 ^+^ memory T cells, and CD25 ^+^ regulatory T (Treg) cells ^23^. Finally, complete deletion of *Stat5a* and *Stat5b* using *Cre-LoxP* approaches demonstrated that STAT5a and STAT5b are absolutely required for lymphocyte development, as *Stat5a/b ^-/-^* mice had profound blocks in lymphocyte development, which mimicked that observed in *Il7r ^-/-^* mice ^24, 25^. These studies definitively demonstrated that the STAT5 hypomorph mice retained significant STAT5 function. Studies with STAT5 knockout mice demonstrated that STAT5 plays a critical role in the development of γδ T cells, as it regulates T-cell receptor (TCR) γ gene rearrangement ^26, 27^. Likewise, STAT5 is required for expansion of double-negative thymocytes ^25^. Finally, IL7R/STAT5 signaling plays an important role in CD8 versus CD4 lineage choice, and increased STAT5 signaling promotes CD8 T-cell differentiation ^28^. The mechanism by which STAT5 regulates early B- and T-cell development is still somewhat unclear, but there is clearly a key role for STAT5 in driving the expression of the pro-survival gene *Mcl1* ^29^. In addition, STAT5 promotes CD8 differentiation by upregulating the transcription factor *Runx3* ^28^. Additional work is required to obtain a more complete understanding of the molecular mechanisms by which STAT5 entrains lymphocyte development.

## STAT5 promotes development of specific T-cell subsets

The availability of both STAT5 gain-of-function and complete loss-of-function mice allowed for a more refined examination of the role of STAT5 in various T-cell subsets. STAT5 was found to play an important role in the development of T helper type 1 (TH1), TH2, TH9, T helper type GM-CSF (TH _GM_), and Treg cell subsets.

## T helper type 1

TH1 polarization is driven by IL12 signaling and T-bet expression leading to production of TH1 cytokines, such as interferon gamma (IFNγ). Naïve T cells, however, do not express the IL12 receptor β2 subunit (IL12Rβ2) and thus are unable to respond to IL12. Early studies observed that T cells deficient in JAK3, the kinase required for STAT5 activation downstream of γc-containing receptors, failed to produce IFNγ under TH1 polarizing conditions ^30^. Furthermore, this study observed that IL2 blockade inhibited TH1 differentiation. Subsequent studies revealed that IL2 signaling, via STAT5 activation, potentiates the TH1 fate by inducing IL12Rβ2 and T-bet expression, thereby allowing the cell to respond to IL12 and polarize toward the TH1 fate ^31^.

**Figure 1.**
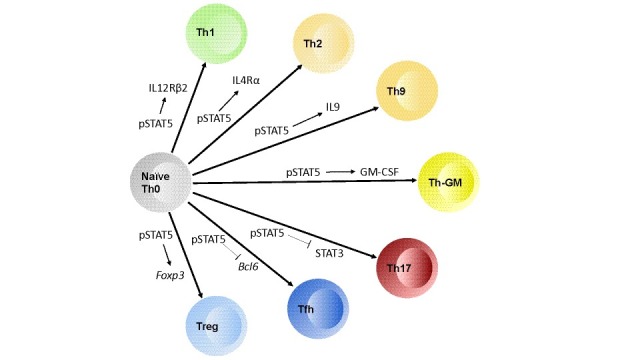
Model outlining how STAT5 activation (pSTAT5) contributes to the differentiation of naïve CD4 T cells into various T helper (TH) subsets. In TH1 development, STAT5 drives interleukin 12 receptor beta 2 subunit (IL12Rβ2) expression. For TH2, STAT5 drives upregulation of IL4Rα. For TH9, STAT5 activation is required for IL9 production. In T helper type granulocyte-macrophage colony-stimulating factor (TH _GM_), STAT5 is critical for granulocyte-macrophage colony-stimulating factor (GM-CSF) production. STAT5 opposes the activation of STAT3, which is required for TH17 differentiation. STAT5 downregulates *Bcl6* expression to inhibit T follicular helper (TFH) cell differentiation, and in regulatory T cells STAT5 turns on *Foxp3* as well as *CD25.*

**Figure 2.**
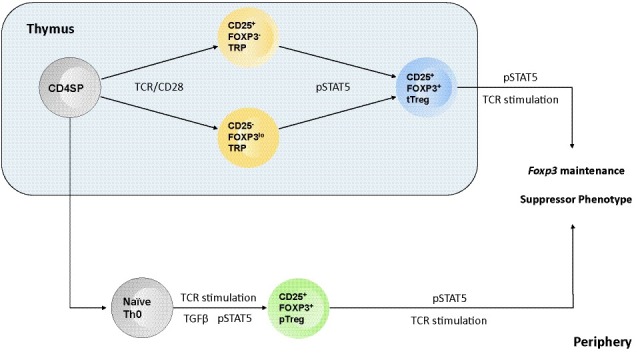
Model outlining the roles STAT5 plays in T regulatory biology in both the thymus and the periphery. STAT5 activation (pSTAT5) is required to complete the differentiation of thymic regulatory T (tTreg) cells and initiate the differentiation of peripherally induced regulatory T (pTreg) cells. STAT5 is also critical for the maintenance of Foxp3 expression, via binding the Cns2 regulatory region in Foxp3, and the suppressor phenotype of regulatory T cells.

## T helper type 2

Similar paradigms have been observed with respect to TH2 polarization, which requires IL4 signaling and GATA3 expression. Early studies hinted at a role for STAT5 in TH2 development as T-cell production of IL4 was diminished without IL2 ^32, 33^. Subsequent studies demonstrated that STAT5 binds to the *Il4* locus and drives IL4 production independently of GATA3; however, GATA3 expression is still critical for the adoption of the TH2 fate ^34– 37^. It was later revealed that STAT5 mediates TCR-induced IL4 receptor alpha (IL4Rα) expression and this role was critical for TH2 induction ^38^. This latter study suggested that STAT5 was induced by IL2 in differentiating TH2 cells. Additional studies have shown that TSLP-dependent activation of STAT5 can also contribute to proliferation, survival, and function of TH2 cells ^39^. In a more recent study, another unique role of STAT5 was observed in TH2 polarization. This study indicated that STAT5 activation drove expression of NLRP3, a component of the inflammasome, in T cells. Moreover, this expression of NLRP3 was required for efficient TH2 polarization, an effect that was due to the ability of NLRP3 to form a complex with IRF4, which in turn induced the expression of TH2 cytokines such as IL4, IL5, and IL13 ^40^. Unlike STAT5 deficiency, however, NLRP3 deficiency did not reduce IL4Rα expression. These studies have illustrated that STAT5 plays a unique role in TH2 development and function.

## T helper type 9

TH9 T cells, a subset closely related to the TH2 lineage, differentiate in the presence of transforming growth factor beta (TGFβ) and IL4 and are defined by prominent IL9 production. Initially, it was observed that the presence of IL4 inhibits TGFβ-driven induction of FOXP3 via a STAT6/GATA3-dependent mechanism ^41, 42^. This initial study found that instead of generating suppressive induced Treg cells, the combination of TGFβ and IL4 formed effector cells that produced IL9 and IL10, and thus resembled TH9 T cells. Thus, much like in TH2 cell differentiation, STAT5 plays a key role in TH9 development and function. The idea that STAT5 plays an important role in TH9 development is supported by the fact that TSLP/STAT5 induces IL9 production, which was required for allergic airway inflammation induced by TSLP ^43^. Consistent with this idea, two recent studies demonstrated that activated STAT5 binds to the *Il9* promoter and facilitates *Il9* transcription by driving an activated chromatin configuration characterized by reduced H3K9 histone methylation ^44, 45^. This effect was reversed by IL21-driven induction of BCL6, which also interacts at adjacent locations in the *Il9* promoter. Subsequent studies demonstrated that IL6-mediated activation of STAT3 opposes STAT5-driven differentiation of TH9 cells; however, this effect was mediated by inhibition of STAT5 activation through diminished IL2 production and not via induction of BCL6 ^46^. Thus, STAT5 activation and pathways that intersect with STAT5 signaling play important roles in TH9 differentiation.

Whereas the precise mechanisms by which STAT5 contributes to specific T helper subset differentiation are unique, the general mode by which STAT5 acts is very similar. Namely, STAT5 functions to prime T cells such that they are competent to respond to the cytokine milieu and differentiate into a particular T helper subset. This suggests a model whereby appropriately activated T cells, receiving TCR and co-stimulation, upregulate IL2 production and via autocrine signaling activate STAT5. Activated STAT5 then induces the expression of polarizing cytokine receptor genes, such as *IL12Rβ2* and *IL4Rα*, allowing these cells to integrate the local cytokines into an appropriate differentiation decision. A similar mechanism may hold for Treg cell differentiation, as STAT5 can upregulate CD25 expression ^47^, which is required for efficient Treg cell differentiation. Furthermore, STAT5 acts in all of these T-cell subsets to drive the expression of T helper subset cytokines. Thus, STAT5 activation plays a crucial role in the differentiation and function of TH1, TH2, and TH9 subsets.

## T helper type GM-CSF

Recently, another unique T helper subset which produces GM-CSF and IL3 was observed: the TH _GM_ subset. A 2014 study observed that TH _GM_ cells were critical mediators of disease progression in a murine model of autoimmune neuroinflammation: experimental autoimmune encephalomyelitis ^48^. This article observed that IL7-driven, not IL2-driven, STAT5 activation is required for the formation of these GM-CSF-producing pathogenic T cells. The authors also provide evidence that TH1 and TH17 differentiation cues are inhibitory to the development of TH _GM_, similar to findings in a human study which observed that IL17 antagonistically regulated GM-CSF-producing T cells that also trafficked to the central nervous system of patients with multiple sclerosis ^49^. Furthermore, the study by Sheng *et al*. showed that the TH _GM_ cells are a unique T helper subset, as their expression profile is distinct from those of both TH1 and TH17 cells ^48^. Interestingly, another study observed that IL2Rα polymorphisms associated with multiple sclerosis potentiated IL2-mediated GM-CSF production in TH cells; however, the production of IFNγ and IL17 was unaffected ^50^. Thus, STAT5 activation has an important role in the development of TH _GM_ cells and may contribute to their pathogenicity in neuroinflammation.

## Regulatory T cells

STAT5 plays a central role in the development and function of Treg cells. Early studies identified CD25, the high-affinity IL2Rα chain, as an accurate marker for suppressor T cells ** ^51^. ** Subsequent studies observed that mice deficient in CD122, the IL2Rβ chain, developed autoimmune disease because they were devoid of functional Treg cells ^52^. Similar results were observed in mice lacking CD25, the IL2Rα chain ^53, 54^, and in human patients with loss-of-function mutations in STAT5b ^55^. These observations suggested that STAT5 activation, downstream of IL2 receptor signaling, was important for Treg cell development or function or both.

## STAT5 in the development of regulatory T cells

Successive studies on the differentiation of thymus-derived Treg (tTreg) cells built on the observation that *Il2rb ^-/-^* mice failed to develop Treg cells. Initial studies demonstrated that STAT5 was the critical downstream effector in IL2Rβ signaling that drove tTreg cell development and that this was due in part to direct targeting of STAT5 to the *Foxp3* promoter ^56– 58^. Follow-up studies proposed a two-step model for tTreg cell development. In step one, high-affinity TCR stimulation drove the expression of CD25 and the development of a CD25 ^+^FOXP3 ^-^ Treg progenitor cell. In the second step, Treg progenitor cells competed for a limiting amount of thymic IL2; progenitor cells that competed effectively for IL2 and activated STAT5 then converted into mature Treg cells ^59, 60^. Consistent with this model, constitutive STAT5 activation was sufficient to drive a subset of conventional thymocytes into the Treg cell lineage ^60^. A more recent study observed that the degree of TCR stimulation a thymocyte received correlated with the expression of the TNFRSF members GITR, OX40, and TNFR2 ^61^. Upregulation of these TNFRSF members facilitated tTreg cell development by sensitizing thymocytes to IL2 stimulation. Those thymocytes with the highest expression of GITR, OX40, and TNFR2 responded to much lower doses of IL2 by activating STAT5 and thus initiating the final step in Treg cell differentiation. Other recent studies have continued to point to the modulation of STAT5 as a critical factor in tTreg cell differentiation. For example, TRAF3 activation dampens tTreg cell development via inhibition of STAT5 activation ^62^. It is not yet clear what regulates TRAF3 in Treg progenitor cells, but one possibility is that GITR/OX40/TNFR2 are involved in direct degradation of TRAF3 and thereby promote increased IL2 sensitivity. Likewise, thymocytes deficient in IFNAR fail to readily develop into tTreg cells. This effect was due to IFNα/IFNβ enhancement of STAT5 activation, either directly or indirectly ^63^. Thus, multiple pathways all impinge on STAT5 in developing Treg cells to regulate the number of Treg cells generated in the thymus.

A distinct type of tTreg progenitor cell population was also recently proposed. These progenitor cells express low levels of FOXP3 but no detectable CD25 (CD4 ^+^CD25 ^-^FOXP3 ^lo^). However, differentiation of this population into mature tTreg cells was still dependent on IL2/STAT5 activation ^64^. A subsequent study suggested that the formation of CD25 ^*-*^FOXP3 ^lo^ tTreg progenitor cells was more dependent on IL15 than the parallel CD25 ^+^FOXP3 ^*-*^ tTreg progenitor subset ^65^. Future studies will need to extend these observations and determine whether there are distinct roles for IL2 and IL15 in tTreg progenitor cell formation and determine whether these effects are also dependent on STAT5 activation.

In addition to tTreg cells, there is another well-accepted class of Treg cells that differentiate from naïve CD4 ^+^ T cells outside the thymus (peripheral Treg, or pTreg, cells). Multiple studies have established that this class of Treg cells is important for maintaining complete tolerance, particularly at mucosal sites interacting with commensal microbes ^66, 67^. Conversion of naïve T cells to pTreg cells is driven by TGFβ ligation; however, this conversion is also dependent on STAT5 activation via IL2 signaling ^68^. IL2/STAT5-dependent signals are required not only for the conversion of naïve T cells into pTreg cells *in vitro* but also to generate these cells *in vivo* ^69^. Further studies indicated that without IL2 the stability of TGFβ-induced Treg cells was greatly diminished ^70^. A more recent study has provided some mechanistic details on the convergence of TGFβ and STAT5 in controlling pTreg cell differentiation. Specifically, hydrogen sulfide is required to activate TET1 and TET2 demethylases and maintain Treg cell homeostasis ^71^. Furthermore, these authors observed that activated SMAD3, downstream of TGFβ signaling, and STAT5, downstream of the IL2 receptor, targeted TET1 and TET2 to the *Foxp3* locus and initiated a hypomethylated state, which facilitated stable expression of *Foxp3* in Treg cells.

## STAT5 in regulatory T-cell function

In addition to its role in the differentiation of both tTreg and pTreg cells, a critical role for STAT5 has been observed in Treg cell maintenance and function. For example, Blazar and colleagues demonstrated that in the context of graft-versus-host disease, Treg cells expressing a constitutively active *Stat5b* transgene provide better protection than wild-type Treg cells ^72^. One proposed mechanism by which STAT5 enhances Treg cell functionality is via binding sites within the *Foxp3* gene locus, functioning to stabilize expression of *Foxp3* and thus the suppressor phenotype. More recent studies have provided support for such a function. Specifically, the *Cns2* enhancer region, which binds several transcription factors, including STAT5, was shown to be required for the maintenance of *Foxp3* expression ^73^. This study further demonstrated that STAT5 binding to *Cns2* enhanced the stability of Treg cells within inflammatory contexts. A subsequent study provided additional evidence that STAT5 plays a central role in maintaining Treg cell homeostasis. First, using histocytometric analysis of whole lymph nodes, the authors observed that Treg cells which contain activated STAT5 are clustered around IL2-producing effector cells that are being stimulated by self-antigen ^74^. Taking this observation further, the authors demonstrated that Treg cells are unable to properly restrain IL2-deficient effector cells and that the IL2-deficient effector T cells had longer interaction times with dendritic cells. This study also provided data that this suppressive function was dependent on TCR signaling in the Treg cells, a conclusion that was supported by a study by Rudensky and colleagues ^75^. To further understand the role of STAT5 activation in mature Treg cells, another study used *Foxp3-Cre* to drive deletion of the IL2Rβ chain in mature Treg cells. Those experiments largely recapitulated the severe autoimmunity observed in *Il2rb ^-/-^* mice ^76, 77^. To understand whether this effect was due to an inability to activate STAT5 or another pathway downstream of the IL2 receptor, the authors generated mice in which a *Stat5b-CA* transgene was integrated into the *ROSA26* locus preceded by a *loxP* flanked *Stop* cassette. Importantly, the *Rosa26-Stat5b-CA* transgene was able to rescue the autoimmune symptoms observed in *Foxp3-Cre x Il2rb ^FL/FL^* mice. Similar to the report by Blazar and colleagues ^72^, this latter study also observed that Treg cells expressing STAT5b-CA were more potent suppressor cells ^76^. Interestingly, RNA-Seq studies comparing wild-type and STAT5b-CA-expressing Treg cells revealed that the STAT5b-CA gene signature was unique and not simply an enhancement of the baseline Treg gene profile. Thus, STAT5 plays a multifunctional role in Treg cell biology. Initially, STAT5 acts as a central effector in initiating the differentiation of Treg cells but, in mature Treg cells, drives their suppressive capabilities and maintains FOXP3 expression. Thus, STAT5 acts as a bridge between effector and suppressor responses, via integration with TCR signaling, to prevent effector responses toward self-antigens while permitting responses to non-self-antigens.

## STAT5 inhibits the development of other T helper subsets

Although STAT5 is required for the development and function of some T helper subsets, it also plays an important role in blocking the development of other T helper subsets, most notably TH17 and T follicular helper (T _FH_) cells. TH17 cells can be generated by stimulation with cytokines that activate STAT3, consistent with a role for STAT3 in TH17 generation ^78– 80^. Subsequent studies demonstrated that the inflammation observed in IL2-deficient mice stemmed from not only a lack of Treg cells but also the fact that IL2 and STAT5 signaling was no longer able to counter the development of inflammatory TH17 cells ^81^. ChIP-Seq studies of STAT3 and STAT5 in CD4 ^+^ T cells showed that these two transcription factors bound to identical sites within the *Il17* gene locus and exerted opposite effects on gene transcription ^82^. Other studies demonstrated that IL2/STAT5 signaling can also affect TH17 development by downregulating expression of the IL6R, which is required to activate STAT3 ^83^. Although the molecular mechanisms by which STAT5 repressed *Il17* transcription have not been completely defined, it appeared that at least three mechanisms could exist. First, STAT5 directly competed with STAT3 for DNA binding and thereby prevented STAT3 from directly inducing *Il17* transcription. Second, STAT5 binding also correlated with binding of the co-repressor NCOR2 and thus might actively repress gene transcription by altering histone methylation or acetylation ^82^. Third, STAT5 repressed expression of the IL6R, leading to reduced activation of STAT3 ^83^. Further studies are needed to clarify the mechanisms by which STAT5 represses or prevents gene transcription.

In addition to suppressing TH17 differentiation, STAT5 inhibits the development of T _FH_ cells. T _FH_ cells require STAT3-inducing cytokines, such as IL21 and IL6, for their differentiation ^84^. In contrast, the STAT5-inducing cytokine IL2 was initially shown to inhibit T _FH_ cell development ^85– 87^. Moreover, these studies demonstrated that the effect of IL2 required STAT5 activation ^18, 86, 87^. This effect appears to involve negative regulation of *Bcl6*, a key transcription factor required for T _FH_ cell differentiation ^18, 88– 90^. STAT5 binds to the *Bcl6* gene promoter and potently blocks *Bcl6* transcription ^18, 91^. The mechanism by which STAT5 prevents *Bcl6* gene transcription remains unclear, although it is possible that this once again involves competition between STAT5 and STAT3 for common binding sites in the *Bcl6* gene. More recent studies have found that IL7 also plays an important role in T _FH_ cell differentiation. These studies demonstrated that TH1 cells which lack IL2R expression eventually upregulate both the IL6R and the IL7R. This results in a bi-potent state, in which cells that are stimulated with IL7 activate STAT5, block T _FH_ cell differentiation, and preferentially give rise to central memory T cells. In contrast, preferential exposure to IL6 induces *Bcl6* transcription via a STAT3-dependent process and promotes T _FH_ cell differentiation ^92^. A subsequent study demonstrated that this also involves additional feedback loops, as BCL6 has been shown to bind to many STAT5 binding sites (including in the *Il7r* gene) in T _FH_ cells and inhibit the expression of these STAT5-dependent genes ^93^.

## Future directions

It is now clear that STAT5 plays important roles in both T-cell development and shaping the CD4 ^+^ T-cell immune response. However, major gaps remain in our knowledge. First, substantial evidence now supports the idea that STAT5 competes for binding sites with opposing effectors (for example, STAT3 and BCL6), but the molecular mechanisms by which STAT5 alters the epigenome to enhance or repress transcription remain unclear. Second, we know that STAT5 can interact with co-activators or co-repressors ^94– 96^, but we do not know whether these known interactors are critical for STAT5 function. Moreover, very little is known about what determines whether STAT5 induces or represses transcription at specific gene loci. One study suggested that this may be due to STAT5 binding as a dimer versus a tetramer ^97^. In contrast, other reports, using STAT5 mutant mice in which STAT5 cannot form tetramers, primarily reported defects in STAT5-dependent gene activation and not repression ^98^. Thus, key future questions will be to resolve the molecular mechanisms by which STAT5 alters chromatin structure and promotes or represses gene transcription and to establish what determinants result in STAT5 promotion versus repression of gene transcription.

## Abbreviations

γc, gamma chain; ChIP-Seq, chromatin immunoprecipitation sequencing; GM-CSF, granulocyte-macrophage colony-stimulating factor; IFNγ, interferon gamma; IL, interleukin; IL4Rα, interleukin 4 receptor alpha; IL12Rβ2, interleukin 12 receptor beta 2 subunit; pTreg, peripheral-induced regulatory T cell; TCR, T-cell receptor; T _FH_, T follicular helper; TGFβ, transforming growth factor beta; TH, T helper; TH _GM_, T helper type granulocyte-macrophage colony-stimulating factor; Treg, regulatory T cell; TSLP, thymic stromal lymphopoietin; tTreg, thymus-derived regulatory T cell.
